# Preparation of Surface-Supported Polylactide Spherical-Cap Particles

**DOI:** 10.1021/acs.langmuir.2c01950

**Published:** 2022-11-17

**Authors:** Barbara Kuśmierz, Kamil Wysocki, Maciej Chotkowski, Ilona Mojzych, Maciej Mazur

**Affiliations:** † Department of Chemistry, University of Warsaw, Pasteura 1, 02-093Warsaw, Poland; ‡ Institute of Genetics and Animal Biotechnology, Polish Academy of Sciences, Postępu 36A, Jastrzębiec, 05-552Magdalenka, Poland

## Abstract

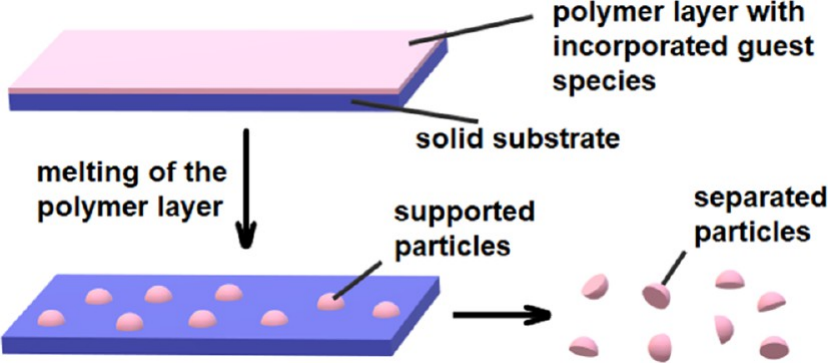

Biodegradable polymer particles are of considerable importance due to their multiple applications in medical diagnostics and therapy. Spherical-cap particles have been prepared in a very general and simple method by melting a thin polymer film supported on a solid substrate that is in contact with a hydrophilic solvent. The melted polymer forms droplets which transform into solid particles attached to the surface after cooling down the sample. This approach has been demonstrated for polylactide adlayers on glass, which, when melted in glycerol, produce an array of polymer particles supported on the surface. The size of the particles depends on the experimental conditions and ranges from tens of nanometers to several micrometers. The particles can be employed to incorporate guest species, for example, drug molecules or inorganic nanoparticles. This has been confirmed herein through entrapment of an anticancer drug (doxorubicin) and radiogold (Au-198) nanoparticles. The resulting structures have been examined using a number of complementary physicochemical techniques including scanning and transmission electron microscopy, atomic force and optical microscopy as well as Raman and fluorescence spectroscopy.

## Introduction

Preparation of biodegradable particles has been a hot topic of research in last several decades. Such particles may find numerous therapeutic or diagnostic applications in medicine, for example, they can be utilized to transport drugs through the body to a specific site or can carry diagnostic tags to allow imaging of pathological tissues.^1,2^

Biodegradable particles can be prepared from a range of materials including synthetic or natural polymers.^3^ Synthetic polymers provide several advantages which include control over their composition and properties. Among the most successful biodegradable polymers are poly-α-esters with short aliphatic chains between ester bonds like polylactide (PLA), polycaprolactone, polyglycolide, and their copolymers. In these macromolecules, the ester bond is relatively easily hydrolyzed under physiological conditions. In consequence, such materials have been employed as prostheses, scaffolds for tissue engineering, or drug delivery vehicles.^4^

Biodegradable polymer particles can be fabricated in a number of methods. One general route is through polymerization of corresponding monomers with the use of hard or soft templates that direct the polymer growth.^5−7^ The second group of methods involves preformed polymers which are assembled into the desired shape or structure. While the list of examples is long, one can mention single^8^ or double emulsion^9^ solvent evaporation method, salting out,^10^ or nanoprecipitation.^11^

The characteristic of the great majority of these methods is that they produce spherical particles. The reason for this is that the surface Gibbs energy of the particle is minimized when the surface area is as low as possible with respect to its volume which is fulfilled for a geometrical ball. To fabricate particles of different shapes, several other approaches have been proposed.^12^ One of the preparative strategies is based on the direct fabrication of nonspherical particles using, for example, microfluidic devices,^13^ electrospraying,^14^ or soft lithography.^15^ For instance, Rolland et al. have developed a soft lithographic method PRINT (particle replication in nonwetting templates) to fabricate submicrometer-sized trapezoidal polymer particles supported on fluorinated silicon substrates through spatially confined polymerization of the corresponding monomers, for example, lactide, triacrylate, or pyrrole. These particles could then be collected through mechanical detachment from the surface.^15^ Nonspherical particles can be also obtained by dewetting a thin polymer layer deposited on a solid support. As a result of the thermodynamic instability of the layer, under the influence of a stimulus (e.g., increased temperature), the film disintegrates to form particles supported on the surface.^16−18^

The other strategy involves the transformation of preformed spherical beads into nonspherical particles through mechanical deformation. For example, in the film stretching method, the spherical particles are embedded in a polymeric material, followed by its mechanical stretching which affects the shape of the incorporated species. Removal of the polymer matrix yields nonspherical particles.^19,20^ Other methods like partial swelling or heating near the glass transition have been also reported.^21,22^

A variety of shapes have been demonstrated including rods,^23^ disks,^24^ cups,^25^ cubes,^26^ and so forth. Due to their anisotropic shape, the particles reveal unusual properties which make them promising in various applications including biosensing, optics, or drug delivery.^12^ For example, hemispherical particles have been shown to reveal enhanced cellular uptake in comparison to spherical beads.^27^ An interesting type of nonspherical colloid is spherical-cap particles. Shelke et al. have shown that such structures form self-assembled aggregates or hyperstructures which exhibit complex dynamical motions in solutions.^28,29^

In the current paper, we propose a new method for the preparation of spherical-cap PLA particles supported on a solid substrate. The structures are fabricated by melting a thin polymer layer that is in contact with a hydrophilic solvent. The incorporation of drug molecules and/or radiogold nanoparticles within spherical-cap particles has also been demonstrated. It is believed that such structures may find applications in medical therapy or diagnostics.

## Experimental Section

### Materials and Methods

#### Chemicals

All chemicals were of the highest quality, commercially available, and used as received: PLA (Noviga, Poland), gold(III) chloride hydrate (reagent grade, Aldrich), doxorubicin hydrochloride (Lancrix), sodium borohydride (Aldrich), NaOH (reagent grade, Chempur), chloroform (POCh, 98.5%), 1-dodecanethiol (Aldrich, ≥98%), hydrochloric acid (37%, reagent grade, Chempur), acetone (POCh, 99.5%), hexane (Aldrich, 95%, anhydrous), and dimethyl sulfoxide (reagent grade, Chempur).

Gold-198 in the form of H^198^AuCl_4_ in aqueous 3 M HCl (activity: 37 MBq/mL, specific activity: 4300 MBq/mg Au) was supplied by POLATOM.

Aqueous solutions were prepared from deionized water (Milli-Q Plus).

#### Instrumentation

Atomic force microscopy (AFM) imaging was performed with a Multimode 5 AFM instrument (Veeco) upgraded to the Multimode 8 version (Bruker). The images have been acquired in the ScanAsyst mode using dedicated silicone cantilevers.

Scanning electron microscopy (SEM) measurements were acquired with a ZEISS MERLIN field emission instrument, while the transmission electron microscopy (TEM) data were recorded using a Zeiss Libra 120 FE TEM. Optical microscopy images (in fluorescence and white light mode) were collected with a Nikon Eclipse LV 100.

Raman spectra and maps were recorded with a Labram HR800 spectrometer (Horiba Jobin Yvon) coupled to an Olympus BX41 microscope. The spectrum was excited with a 532 nm laser. The same instrument was also used to record the fluorescence spectra of doxorubicin.

The activity of Au-198 was determined with an HPGe detector (Canberra, XtRa coaxial detector, efficiency: 40%) using the photo peak at energy 411.8 keV.

Contact angle measurements were performed with a homemade instrument consisting of an optical microscope and a camera.

A spin coater from Laurell Technologies Corporation, model WS-65OSZ-6NPP/A2, was used to fabricate thin polymer films on glass slides.

### Preparative Procedures

#### Preparation of PLA Particles Supported on a Glass Substrate

PLA solutions in chloroform (0.1–3% w/w concentration, 1 mL) were spin-coated onto 24 mm × 24 mm glass slides (spinning time: 1 min, speed: 2000 rpm). Next, the substrate was submerged in glycerol in a glass vessel and heated at 180 °C for 4 min. The sample was then allowed to cool down to room temperature, removed from glycerol, rinsed with deionized water, and dried under a stream of air.

#### Preparation of Au-198-Doped Gold Nanoparticles

Gold nanoparticles doped with Au-198 radioisotope were prepared following the modified procedure reported elsewhere.^30^ 100 μL of ^198^AuCl_4_^–^ (5.589 MBq) in aqueous 3 M HCl was added to a glass vial and evaporated under reduced pressure. Then, 2.40 mL of water and 25 μL of ^197^AuCl_4_^–^ (50 mM) in 50 mM aqueous HCl were added to the vial. While the solution was rapidly stirred with a magnetic stirrer, 150 μL of NaBH_4_ (50 mM) in aqueous NaOH (50 mM) was added with a pipet (which was associated with a rapid change of the color of the reaction mixture). The stirring was continued for 1 min.

The nanoparticles were next transferred to the organic solvent. 1 mL of the nanoparticle solution was mixed with 1 mL of acetone. Then, 2 mL of 4 mM dodecanethiol in hexane was added and vortexed for 30 s. The hexane phase was collected, and the solvent was evaporated under reduced pressure. Finally, the solid residue was dispersed in 4 mL of 0.5% (w/w) PLA in chloroform, yielding a nanoparticle concentration of *ca.* 24.6 μg/mL (*ca.* 359 kBq/mL).

#### Incorporation of Doxorubicin or Gold Nanoparticles in Spherical-Cap Particles

The preparation of PLA particles with incorporated guest species was essentially the same as described above for neat particles. The only difference was that the corresponding PLA chloroform solution contained doxorubicin or gold nanoparticles. To obtain the doxorubicin solution, 50 μL of 1 mM doxorubicin in dimethyl sulfoxide was added to 1 mL of PLA or PLA/AuNp chloroform solution (vide supra). These solutions were subsequently used for spin coating.

## Results and Discussion

**Scheme 1:**
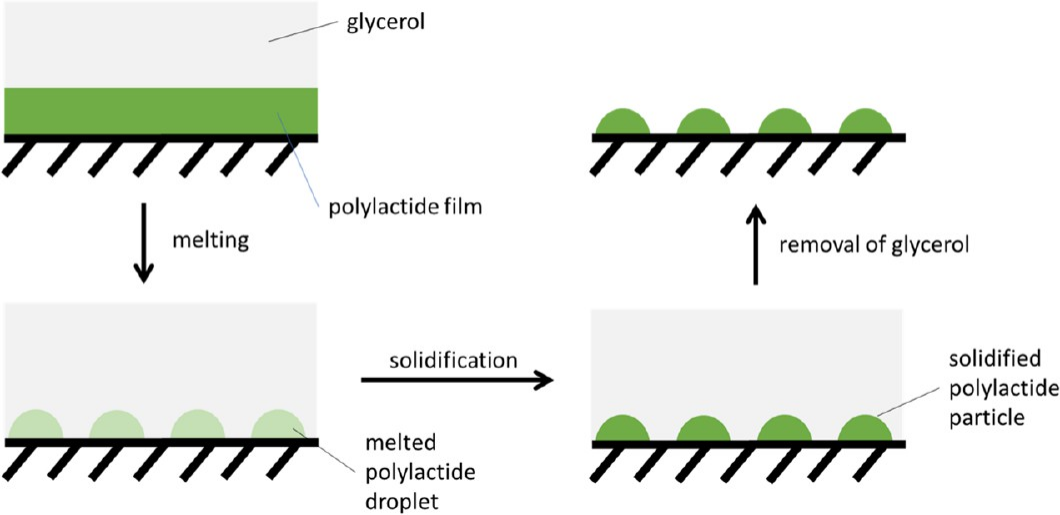
Preparation of Solid-Supported Spherical Cap Particles

The current paper focuses on the development of a novel method for the preparation of spherical-cap polymer particles supported on a solid substrate. The general idea of the preparative process is presented in Scheme 1.

First, a thin layer of polymer is fabricated on the surface of a flat substrate. Then, the polymer layer is melted to yield polymer droplets by increasing the temperature. Finally, the temperature is decreased to solidify the droplets and form the solid-supported particles.

**Figure 1:**
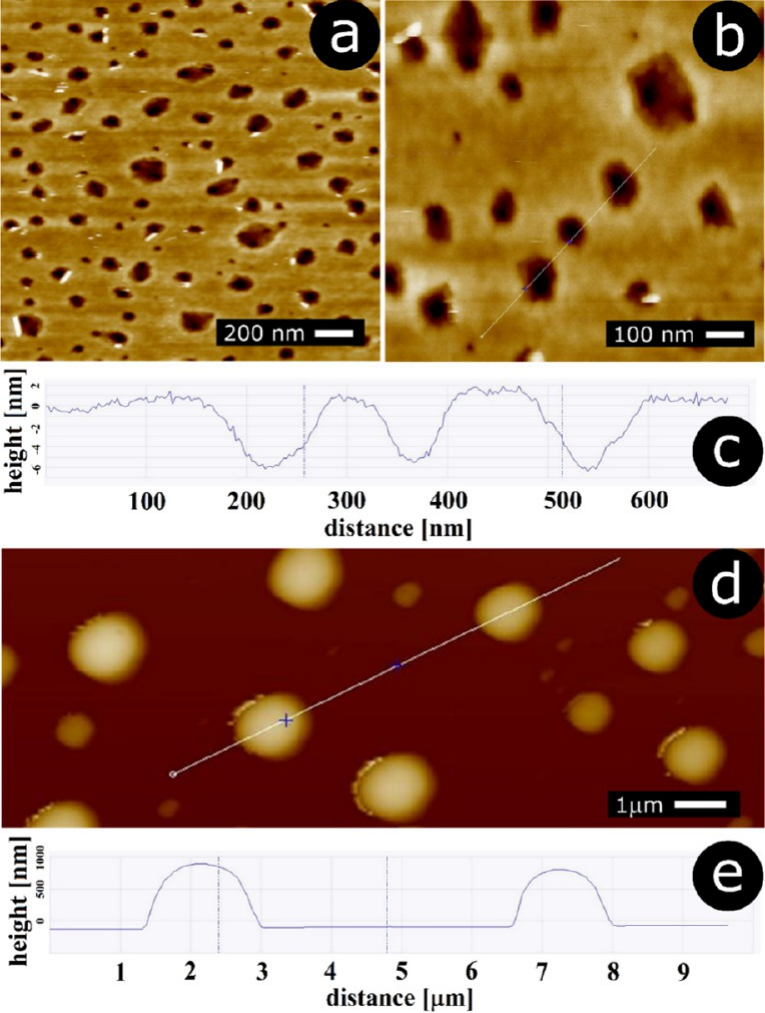
AFM: (a) PLA film spin-coated from 0.5% chloroform solution—lower magnification, (b) PLA film spin-coated from 0.5% chloroform solution—higher magnification, (c) cross-sectional profile through the image shown in b panel, (d) PLA spherical cap particles generated through melting the polymeric film, (e) cross-sectional profile through the image shown in d panel.

In our experiments, we have chosen PLA, which is a well-known, biodegradable, nontoxic, and biocompatible polymer. The PLA layer has been prepared on the surface of a glass slide through spin coating of a 0.5% polymer solution in chloroform. Shown in Figure 1a,b are the AFM images of the polymeric film.

One can see that even though the adlayer is generally smooth, there are numerous holes within its structure, *ca.* 70 nm in diameter (the histogram demonstrating the hole diameter distribution is shown in Figure S1, Supporting Information). A cross-sectional profile through the image is shown in Figure 1c. The depth of the holes is *ca.* 5 nm. It seems that this value can be identified as the thickness of the polymer layer. We were interested further in whether melting the polymer layer induces any morphological changes. The film-coated glass was annealed at 180 °C, which is above the melting point of PLA (*ca.* 155 °C) for 4 min, then cooled down and imaged again with AFM. As can be seen in Figure S2 (Supporting Information), the polymer film is even more smooth as the number of holes is significantly lower.

It is instructive here to calculate the spreading coefficient (S) for this system, which informs whether the layer covering the substrate is stable or is subject to dewetting (dewetting is a spontaneous process where a film on a surface disintegrates into an array of separated objects, for example, droplets or particles)^31^
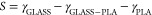
1where γ_GLASS_ is the surface energy of glass, γ_GLASS–PLA_ is the interfacial tension between glass and PLA, and γ_PLA_ is the surface tension of (melted) PLA.

Substituting the formula for γ_GLASS–PLA_ from the Young equation^31^ one gets
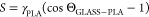
2where Θ_GLASS–PLA_ is the contact angle of molten PLA on glass.

Taking into account that the surface tension of the molten PLA at 180 °C is 26.0 mN/m^32^ and substituting the experimentally determined value of the contact angle (Θ_GLASS–PLA_ = 21°), we obtain the value of the spreading coefficient *S* = −1.73 mN/m.

The obtained value is slightly negative and indicates a relatively low susceptibility of the polymer layer to dewetting. In fact, *S* depends on the contact angle, which may actually be even lower than the determined value, due to the high viscosity of the molten polymer (thus, the spreading coefficient might be closer to zero). On the other hand, the presence of holes in the layer (both before and after annealing) indicates a certain instability of the polymer. However, this does not result in observable rupture of the film and generation of polymer droplets on the substrate surface (at least under the applied experimental conditions).

The abovementioned discussion clearly shows that to achieve dewetting of the melted layer to form liquid polymer droplets (and then to form surface-supported particles), one can change the melting conditions. Thus, we have chosen glycerol, a hydrophilic medium which, being in contact with the polymer, could induce the dewetting of the melted layer. The boiling point of glycerol is 290 °C, which is considerably above the melting point of the polymer. Thus, the PLA film being in contact with glycerol could be melted without boiling the solvent.

Similarly, as in the previous case, it is possible to calculate the spreading coefficient for this system to predict whether the thermodynamic conditions for the dewetting process have been met. For this purpose, one can use a modified formula for *S*, corresponding to the case where the polymer adlayer is in contact with a solvent (here: glycerol)^33^
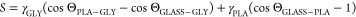
3where γ_GLY_ is the surface tension of glycerol, γ_PLA_ is the surface tension of (melted) PLA, Θ_PLA–GLY_ is the contact angle of glycerol on melted PLA, Θ_GLASS–GLY_ is the contact angle of glycerol on glass, and Θ_GLASS–PLA_ is the contact angle of molten PLA on glass.

Using the determined values of the contact angle (Θ_PLA–GLY_ = 86°, Θ_GLASS–GLY_ = 32°, and Θ_GLASS–PLA_ = 21°) and literature data of surface tension at 180 °C (γ_GLY_ = 47.5 mN/m^34^ and γ_PLA_ = 26.0 mN/m),^32^ one gets *S* = −36.97 mN/m. This value is significantly low as compared to melting the polymer without glycerol. It indicates a high thermodynamic instability of the system and consequently a strong tendency to dewetting. The low value of *S* is influenced by the first term of the eq 3, in particular, the high value of the contact angle of glycerol on molten PLA (the determination of the contact angle was experimentally difficult; however, due to the high viscosity of the molten polymer, which makes it possible to apply a drop of glycerol on it, the obtained value seems to be reliable).

**Figure 2:**
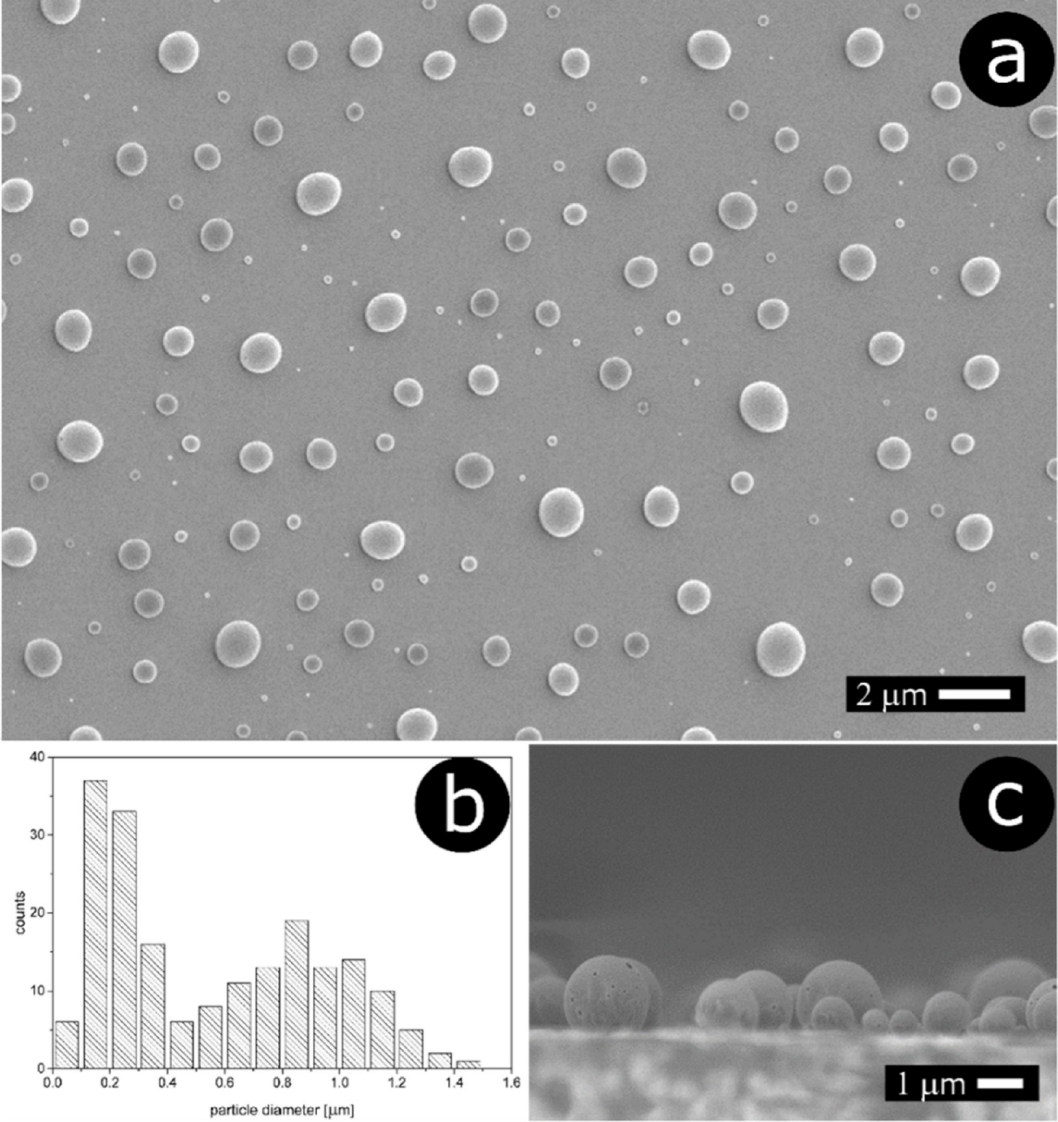
Spherical cap particles generated through melting the polymer film spin-coated from 0.5% solution: (a) SEM image, (b) histogram of particle diameters, and (c) SEM image captured from the side.

Undoubtedly, however, the best way to confirm the accuracy of the abovementioned discussion is experimental confirmation. Therefore, the polymer-coated glass slide was heated in glycerol at 180 °C, followed by cooling down to room temperature. The AFM image and corresponding cross-sectional profile (Figure 1d,e) show that the morphology of the polymeric deposit was completely changed. One can see spherical-cap particles attached to the substrate surface. It appears that the introduction of glycerol to the system promotes dewetting of the liquid polymer film and the formation of polymer droplets. Then, decreasing the temperature below the melting point of the polymer yields solid particles. The SEM image of the structures is shown in Figure 2a. It confirms the results of AFM imaging. The histogram of the particle diameters is shown Figure 2b. One can see bimodal distribution with two populations of particle sizes: larger *ca.* 900 nm and smaller *ca.* 220 nm. A SEM image recorded from the side (Figure 2c) unequivocally confirms that the particles reveal a spherical-cap shape.

The abovementioned data suggest that the final geometry of the spherical-cap particles depends on the interactions at interfaces between liquid polymer, glycerol, and glass. When the polymer solidifies, it retains the shape of the liquid droplet. Based on the SEM data (Figure 2c), one can estimate the contact angle of the polymer droplets on the glass surface (submerged in glycerol). The analysis of the image yields a value of *ca.* 110°. The contact angle can be also calculated from the Young equation
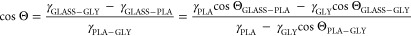
4

Substituting the experimental and literature data into the equation (vide supra) yields a contact angle of 135°, which is somewhat higher than the experimental value.

**Figure 3:**
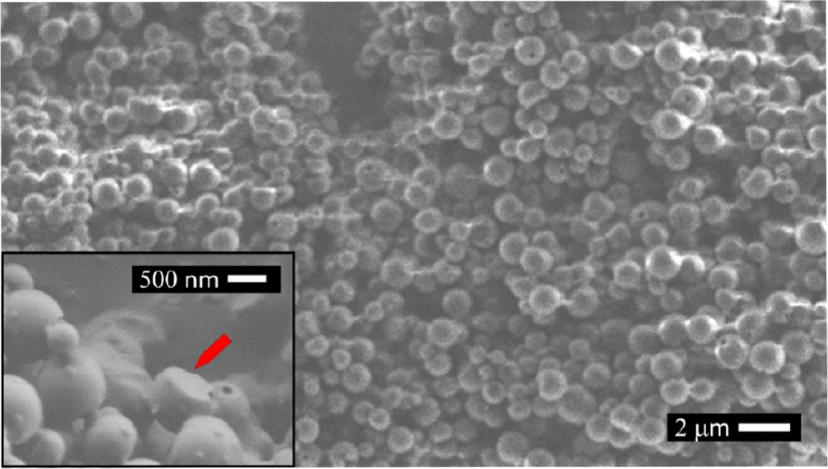
SEM image of spherical cap particles detached from the surface. Inset: magnified image; the arrow depicts the flat interface of the particle that has been in contact with the glass surface.

The spherical-cap shape of the polymer particles can be further confirmed after their detachment from the surface. For this purpose, the particle-decorated slides have been subjected to ultrasound radiation in water, and then, the separated beads have been collected and imaged with SEM (Figure 3). On the magnified image (inset to Figure 3), one can easily identify the flat interface that has been in contact with the glass surface (marked with an arrow).

**Figure 4:**
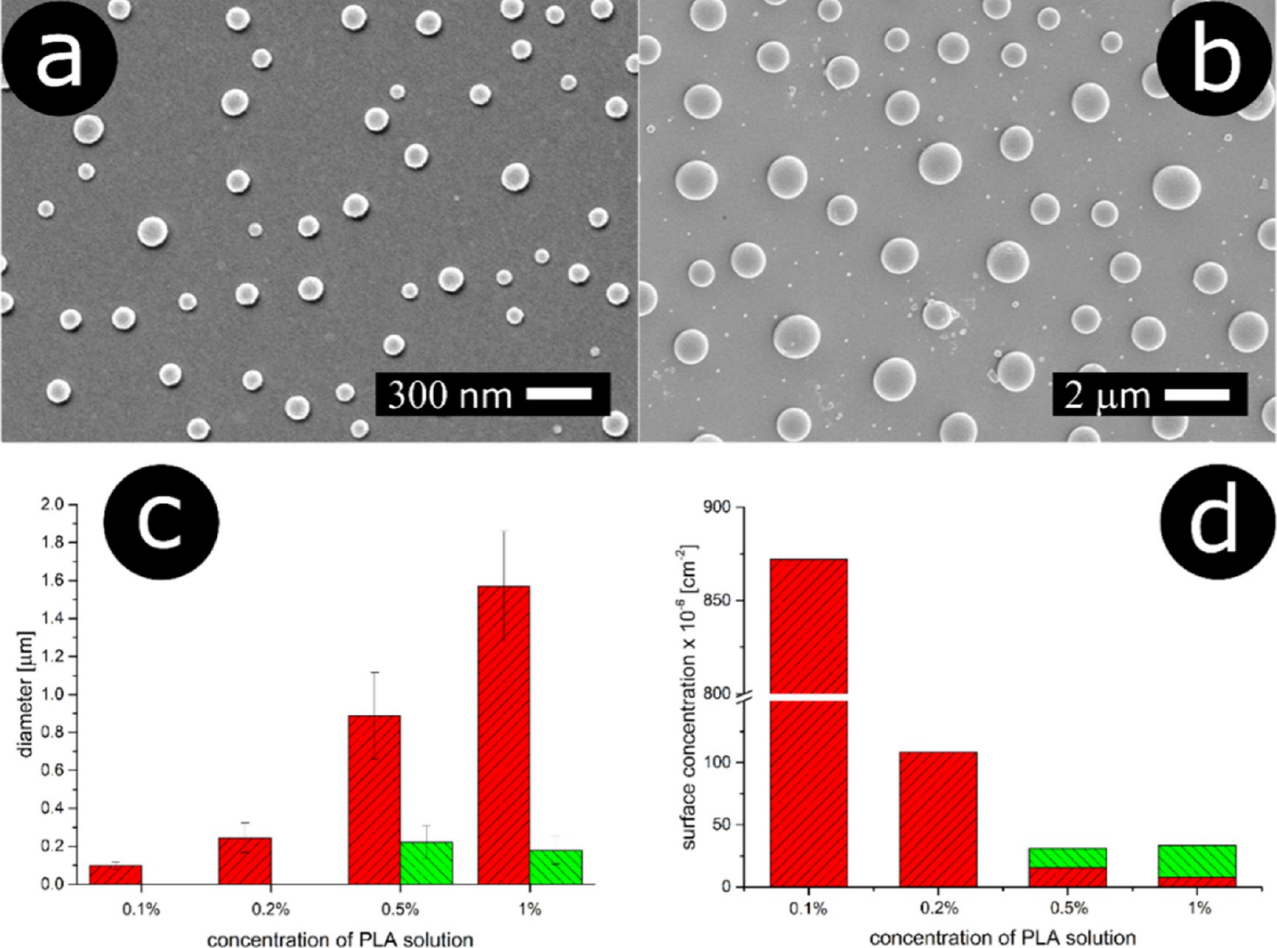
Spherical cap particles generated through the melting of polymer film, spin-coated from (a) 0.1% solution and (b) 1% solution. (c) Histogram showing the average particle diameter for the samples prepared from 0.1 to 1% solutions; for the concentrations of 0.1 and 0.2%, monomodal distribution is observed; thus, the bars (red color) depict the average diameters of the particles. For the concentrations of 0.5 and 1%, one observes bimodal distributions; thus, the average diameters of smaller and larger populations are represented as red and green bars, respectively. (d) Histogram showing the particle surface concentrations for the samples prepared from 0.1 to 1% solutions; for the concentrations of 0.1 and 0.2%, monomodal distribution is observed; thus, the bars (red color) depict the surface concentration of the particles; for the concentrations of 0.5 and 1%, one observes bimodal distributions; thus, the surface concentrations of smaller and larger particle populations are represented as red and green bars, respectively.

The question that can be raised here is whether the number and size of the surface-supported particles can be controlled by modifying the experimental conditions. To answer this question, we have prepared the samples in essentially the same way but changing the initial concentration of PLA. Shown in Figure 4a,b are SEM images of the particles prepared using 0.1 or 1% PLA solution. In both cases, one can see spherical particles uniformly distributed on the surface but of significantly different sizes. For 0.1%, the average particle diameter is 100 nm, while for the higher concentration, it is 1.57 μm (one can also distinguish a population of smaller particles, *ca.* 180 nm in diameter, vide infra). This demonstrates that by increasing the polymer concentration ten times, the size of the particles is scaled 1 order of magnitude. We examined in more detail the effect of the initial polymer concentration on the diameter of the resulting structures by plotting the corresponding histograms based on the SEM data (Figure S3, Supporting Information).

For 0.1 and 0.2%, the histograms reveal monomodal distribution; however, with the increase of the concentration (0.5, 1%), the distributions become bimodal. This trend is shown in Figure 4c. The size of the particles increases monotonically, but starting from the 0.5% concentration a population of smaller particles emerges (*ca.* 200 nm). On the other hand, the surface concentrations of the resulting particles decrease with the increase of the initial polymer concentration in the solution (Figure 4d).

This observation can be explained as follows. When a small concentration of PLA is applied onto a spinning substrate, the thickness of the resulting polymer film is small. In consequence, the amount of polymeric material that is accessible to form particles is low; thus, the size of the particles is also small. With the increase in the concentration, the initial polymer layer is thicker, resulting in considerably larger droplets (particles). However, it appears that when large polymer droplets are produced, occasionally some polymeric material remains on the surface (outside of the large droplets), and this material forms smaller droplets. As a result, a bimodal distribution of particle size is observed. Such a mechanism assumes the immediate melting of the polymer and simultaneous formation of the polymer droplets.

An alternative possible explanation is that the thermally induced dewetting process starts from the hole sites (the presence of holes is shown in the AFM data, vide supra). These holes gradually grow, which is associated with the formation of rims at the growth front. The rims may create filaments or strings, which then break into droplets.^35^ It is possible that for higher polymer concentrations (of solution used for spin coating), larger particles are generated from the nodes of the filament network, while smaller ones are produced by rupture of the filaments, thus yielding bimodal distribution. Even though such a mechanism is possible, as no polygonal patterns of polymer particles are observed,^35^ this may rather suggest the first scenario where the instantaneous breakup of the film is more likely.

**Figure 5:**
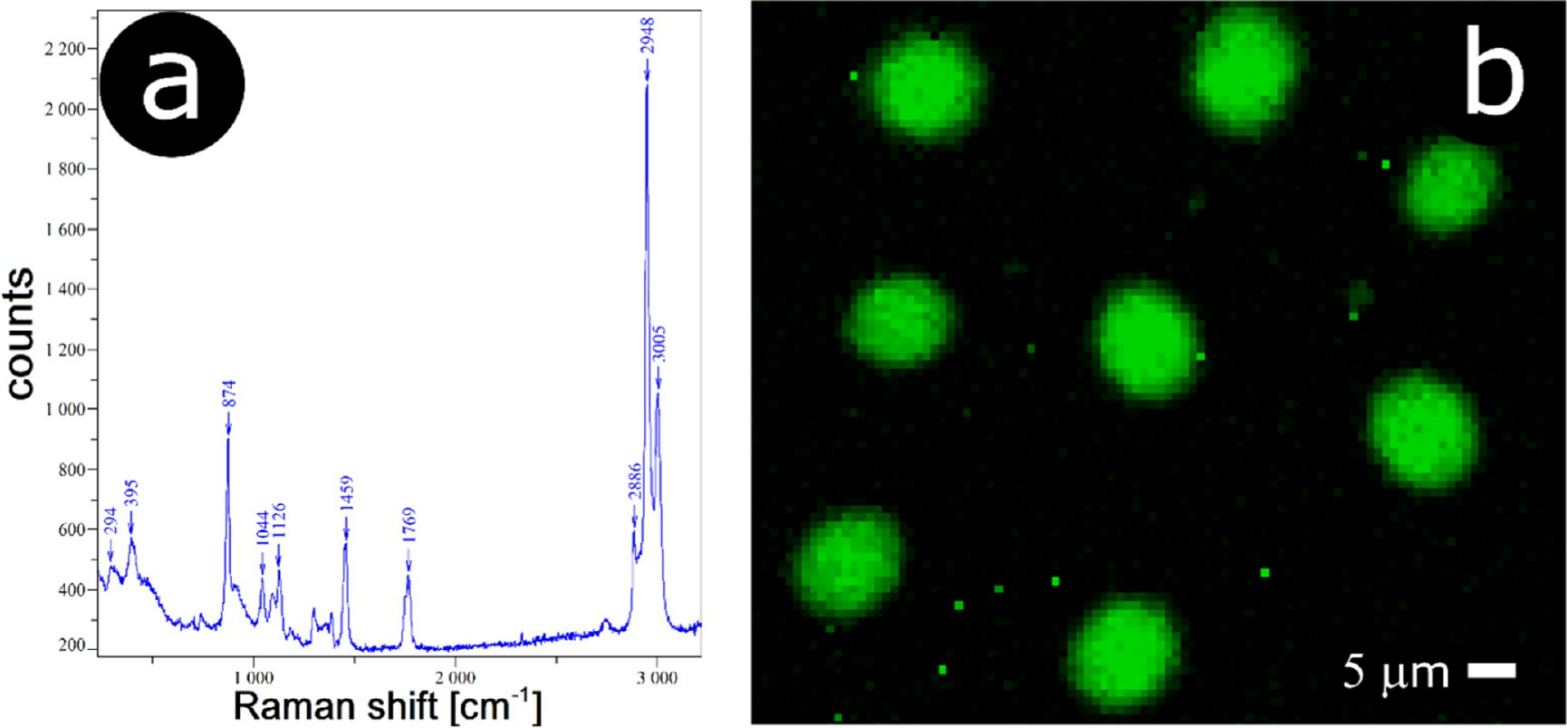
Spherical cap particles supported on a glass substrate: (a) Raman spectrum and (b) Raman map (distribution of 1459 cm^–1^ band).

Regardless of the exact mechanism of polymer dewetting, finally, the substrate surface becomes decorated with polymer particles, while the majority of the surface area is left not covered with the polymer. To confirm this notion, we used Raman microscopy (the sample has been prepared from 5% PLA solution which produces large enough polymer particles). The laser beam was focused onto an individual polymer particle, and the Raman spectrum was recorded (Figure 5a). The spectrum reveals all the bands characteristic of PLA.^36^ Specifically, the bands at 2886, 2948, and 3005 cm^–1^ are assigned to C–H stretching vibrations. A strong mode at 1769 cm^–1^ is attributed to the C=O stretching vibration. The asymmetric methyl deformation mode is seen at 1452 cm^–1^ as an intense Raman line. Several other bands characteristic of PLA for example 1126, 1044, 874, and 395 cm^–1^ (*r*_as_CH_3_, νC–CH, νC–COO, and δCCO, respectively) are also seen in the spectrum. On the other hand, when the laser beam is focused on the substrate surface not occupied by the particles, no bands of PLA are observed, only weak signals attributable to glass (the spectrum not shown).

Next, we recorded the Raman map as a distribution of the 1459 cm^–1^ band (Figure 5b). One can see that the intensity in the Raman signal reflects the morphology of the polymeric structures. No signal attributable to PLA is observed in the areas outside of the particles.

The abovementioned discussion clearly shows that by simple control of the temperature and appropriate selection of the contacting liquid medium, one can easily fabricate an array of semispherical particles supported on a solid substrate. The question that arises here is whether it is feasible to incorporate guest species within the formed polymer particles. Such a task would be important from the point of view of possible medical applications, for example, in the encapsulation of drugs. To test this possibility we have used doxorubicin, which is a well-known chemotherapeutic agent that exhibits intrinsic fluorescence.

The incorporation of guest molecules is possible at the stage of preparation of polymeric particles. The drug is added to the polymer solution prior to spin coating. In consequence, a thin polymeric film that contains the drug is formed. Next, the layer is melted to produce an array of spherical-cap particles with incorporated molecules. Taking into account the initial concentrations of the polymer and the drug in the solution used for spin coating and based on a simple calculation, one can estimate that the loading density of the drug is *ca.* 3.77 mg per gram of PLA (for 0.5% PLA concentration).

**Figure 6:**
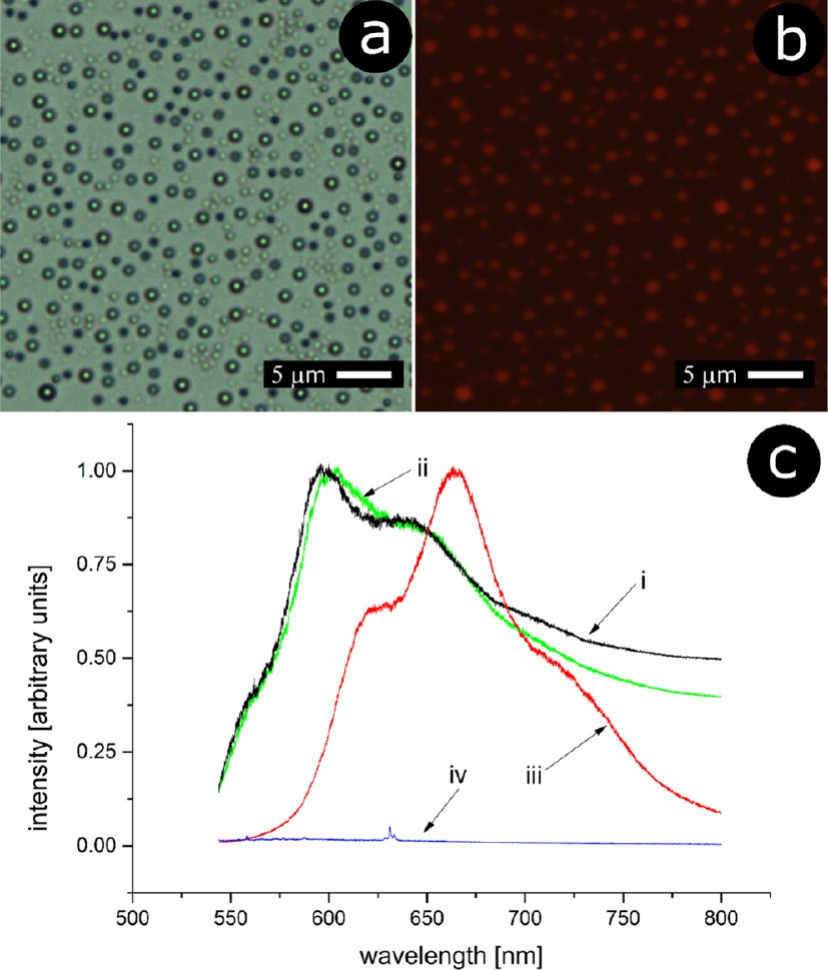
Supported spherical cap particles with incorporated doxorubicin: (a) optical image under white-light illumination; (b) fluorescence microscopy image; (c) fluorescence spectra: (i)—doxorubicin incorporated in PLA cap particles, (ii)—doxorubicin incorporated in the PLA thin film, (iii)—doxorubicin hydrochloride in the powder form, and (iv)—reference spectrum of PLA (without incorporated doxorubicin).

The particles have been examined with fluorescence microscopy. Shown in Figure 6b is a microscopic image of PLA beads with incorporated doxorubicin (an optical image under white-light illumination is also included for comparison, Figure 6a). One can see that the fluorescence signal matches the morphology of the sample: the emission is observed from the particles, while no signal is seen outside of the structures.

The corresponding fluorescence spectrum of doxorubicin contained in the PLA particles is shown in Figure 6c, spectrum (i) [as a reference, the spectrum of PLA without incorporated doxorubicin is also shown, spectrum (iv)]. The spectrum exhibits the emission band with a maximum at 598 nm (two additional overlapped bands are also seen at *ca.* 560 and 640 nm), which is in accordance with the literature data for doxorubicin encapsulated in polymeric particles (e.g., in PLGA nanoparticles) or in the solution.^37,38^ Interestingly, the spectrum of crystalline doxorubicin hydrochloride (in the powder form) exhibits the emission bands that are red-shifted by *ca.* 70 nm [Figure 6c, spectrum (iii)]. This indirectly confirms that doxorubicin must have been incorporated within PLA particles, as its spectrum differs from that of the nonincorporated drug. One should also note that heating the sample to 180 °C (to melt the polymer) does not affect the incorporated doxorubicin (the melting point of doxorubicin is 195 °C; above this temperature, it can be gradually decomposed)^39^ as its emission band is only slightly blue-shifted with respect to the spectrum of doxorubicin contained in the PLA film before heating the sample [Figure 6c, spectrum (ii)]. It seems this shift may be due to the change in the immediate environment of the drug molecules during polymer melting.

**Figure 7:**
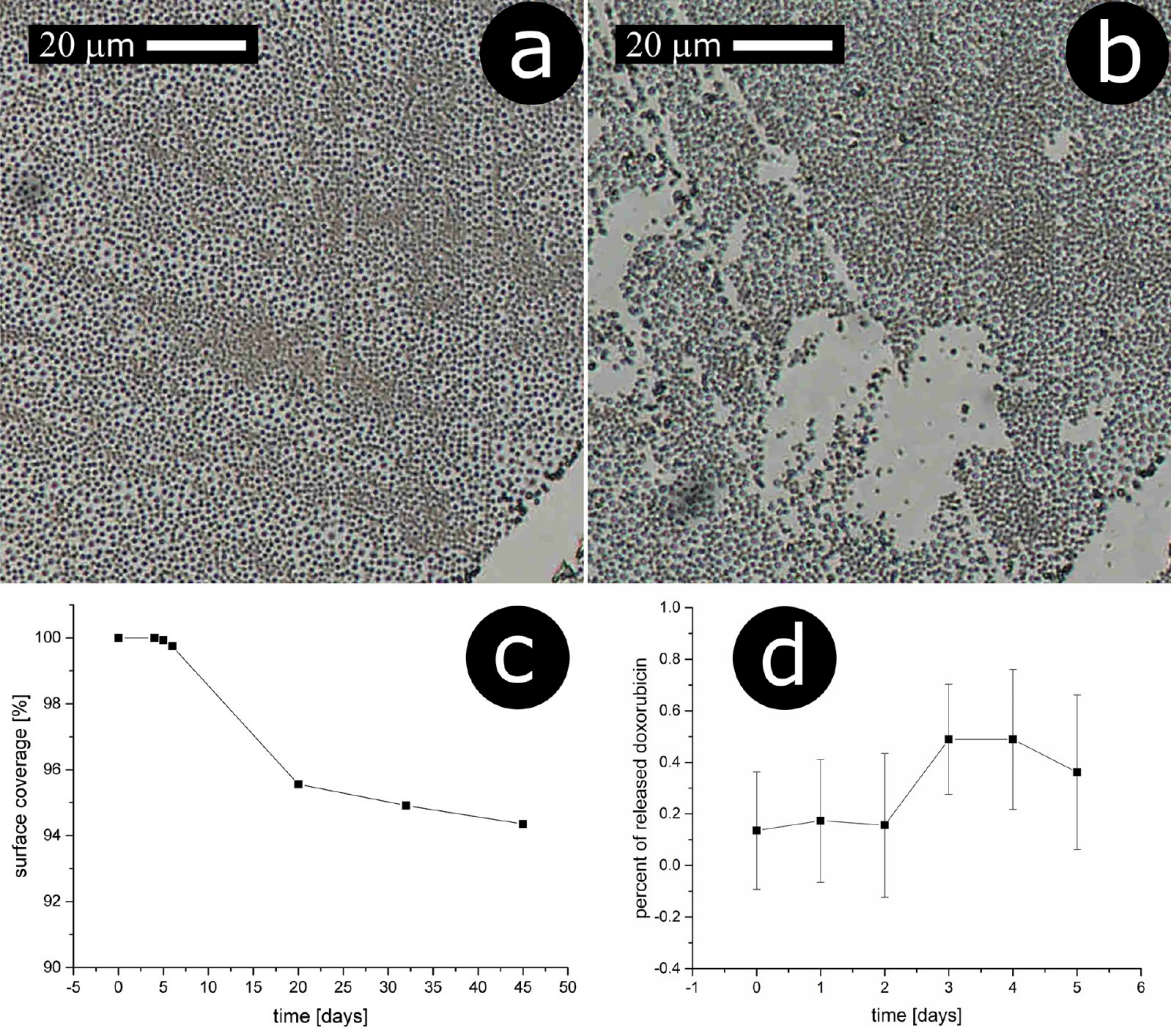
Incubation of solid-supported PLA beads with incorporated doxorubicin in pH 7.4 buffer solution at 37 °C: (a) microscopic optical image after 5 days of incubation, (b) microscopic optical image after 45 days of incubation, (c) dependence of surface coverage with incubation time, (d) percentage of released doxorubicin vs incubation time. In microscopic images, a scratch is seen in the lower right corner, which was made to facilitate finding a given place on the sample.

The next question that arises here is the stability of the particles supported on glass: whether they remain attached to the surface for a prolonged time and whether they may release the incorporated drug molecules. To answer this question, the glass substrate decorated with doxorubicin-containing particles was incubated in pH 7.4 buffer solution at 37 °C for several days. The temperature and pH were selected to mimic the conditions in the human body. The samples have been examined with optical microscopy under white-light illumination. Shown in Figure 7a,b are optical images of particle-decorated glass slides after incubation for 5 and 45 days, respectively. While the coverage with the particles is close to 100% for the sample incubated for 5 days, one can see several noncovered areas of the surface after 45 days. This shows that some of the particles have been detached from the surface.

Based on the analysis of the microscopic images, we have plotted the dependence of surface coverage with incubation time (Figure 7c). One can see that the surface coverage starts to decrease after *ca.* 5 days reaching a coverage of *ca.* 94% after 45 days. Since, for the first 5 days, we see practically no detachment of the particles from the surface, we have examined whether within this time period one could observe any release of doxorubicin to the contacting solution. The slide decorated with the doxorubicin-containing particles was placed in a cuvette poured with a buffer solution, and fluorescence of this solution at 600 nm was monitored with time (the data are presented as a percentage of released doxorubicin)—Figure 7d. One can see that the amount of doxorubicin released directly to the aqueous phase is well below 1%; thus, it can be assumed to be negligible.

The important conclusion from the abovementioned experiments is as follows. The array of PLA particles supported on the solid substrate may be used for the incorporation of guest species; however, it appears that they are generally not secreted to the contacting medium. On the other hand, for a longer incubation time, the particles themselves are detached from the surface carrying their payload. It can be speculated that they finally may release the incorporated molecules, but this likely takes much more time as it requires the degradation of the polymeric matrix.

**Figure 8:**
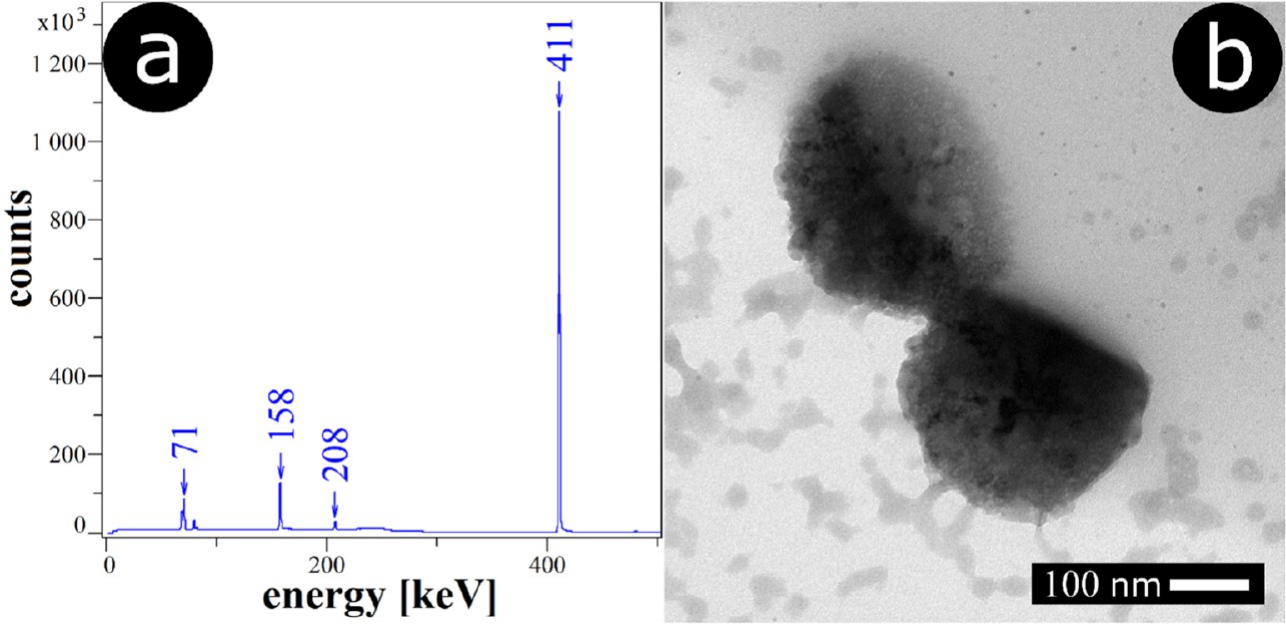
a) γ-ray spectrum of PLA particles with incorporated radiogold nanoparticles, (b) TEM of separated PLA particles with incorporated radiogold nanoparticles.

Since the incorporation of guest species appears to be a universal phenomenon, we have tested whether it could be used to incorporate inorganic nanoparticulate species. The nanoparticles have been prepared through the reduction of the AuCl_4_^–^ precursor with sodium borohydride in a biphasic system. For better detection (after incorporation in PLA), the gold nanoparticles (AuNPs) have been doped with the radioactive Au-198 isotope. Au-198 is a β^–^ emitter with a half-life of *ca.* 2.7 days. This radioisotope decays in 98.99% of cases to the first excited state of Hg-198, which then relaxes by emitting γ photon of energy 411.8 keV.^40^ Thus, through the measurements of γ radiation, one can detect the presence of the radioisotope. The prepared AuNPs have been added to the polymer solution followed by the preparation of PLA film on a glass slide and melting at 180 °C in glycerol. Shown in Figure 8a is the γ-ray spectrum of the sample. One can see the peaks at 411 keV (γ) and 71 keV (X-rays) which are attributable to the emission of Au-198. Considerably smaller signals at 158 and 208 keV are also detected in the spectrum. These peaks can be assigned to Au-199 nuclide—the second radioisotope of gold generated during neutron irradiation of stable Au-197, which is apparently present in the sample, even though its abundance is much lower than Au-198. The most important conclusion from these data is that they confirm the incorporation of gold into PLA particles.

One can hypothesize that during the melting of the PLA film, the nanoparticles might be transferred to the solvent (glycerol) which would result in a decrease in their entrapment efficiency in the polymer structures. To test whether this may be the case, the activity of the samples before and after melting has been acquired. The recorded activity of the unmelted sample was 97.8 Bq·cm^–2^, while that after melting was 89.9 Bq·cm^–2^. This result confirms that *ca.* 92% of the nanoparticles remain incorporated in the forming PLA beads, while only 8% seem to be transferred into the glycerol phase. A similar experiment has been performed for the concurrent incorporation of gold nanoparticles and doxorubicin. Both species have been added to the polymer solution, followed by the preparation of polymer particles. For the melting process, we again observe comparable activity loss of *ca.* 5% associated with the transfer of the nanoparticles to glycerol solvent. Assuming the initial concentration of gold nanoparticles in the polymer solution (used for spin coating) and the determined activity loss (from the radiometric experiment), based on a simple calculation, we may roughly estimate the loading of gold in PLA particles at 9.85 mg per gram of PLA (for 0.5% PLA concentration).

Next, the hybrid particles have been detached from the glass slide with ultrasound and imaged with TEM. Shown in Figure 8b is the TEM image of the PLA particles with incorporated gold nanoparticles. One can see a spherical-cap shape of the polymer structures with resolvable darker spots. These spots can be attributed to embedded gold nanoparticles. This additionally confirms that gold nanoparticles became entrapped in the polymer particles.

The use of active gold nanoparticles and radiometric measurements was employed to estimate the yield of encapsulation. However, the polymer particles modified with radiogold nanoparticles are interesting also from the point of view of their potential medical applications. The γ emission can be used to detect Au-198-containing particles in the body with a γ camera or SPECT. On the other hand, the β emission provides the possibility to destroy cancerous cells. From this perspective, the concurrent incorporation of doxorubicin and radiogold nanoparticles in PLA structures is especially interesting. The joint action of β radiation and chemotherapeutic agents may result in synergistic effects, which could be applicable in cancer therapies. Our previous studies have shown the possibility of encapsulation of ^198^AuNP with isothiocyanates^30^ or preparation of radioactive gold composite bioparticles with doxorubicin^41^ to demonstrate synergistic cytotoxic effects in vitro and show perspectives of medical imaging.

## Conclusions

A novel method for the fabrication of spherical-cap particles has been proposed. It is based on the preparation of a thin PLA film on a flat substrate, followed by melting the polymer in contact with a high boiling point solvent (glycerol). The sample is then cooled down to room temperature which yields an array of particles attached to the substrate surface. The particles reveal a spherical-cap morphology which resembles the shape of liquid polymer droplets.

The main novelty of this work is the use of a properly selected solvent to force the dewetting of the polymer. The PLA film on glass in the air is relatively stable and does not disintegrate during the melting of the polymer. To change the stability of the layer, a liquid phase is added deliberately to the system. This affects the thermodynamic equilibrium in the system. Consequently, the polymer dewets to form droplets/particles supported on the substrate surface.

The number and size of the particles can be controlled to some extent by adjusting the polymer concentration of the solution used for spin-coating. For low concentrations, one can produce a large number of small (*ca.* 200 nm) beads, while with the increase of the concentration, two populations of larger (micrometer-sized) and smaller (nanometer-sized) particles are formed.

The spherical-cap structures can act as carriers of drugs or diagnostic tags. Specifically, concurrent incorporation of doxorubicin and Au-198-doped gold nanoparticles may be potentially applied in cancer treatment or diagnostics. The incorporation of guest species seems to primarily involve their mechanical entrapment within the polymer matrix. The degradation time of PLA used in this work is quite long; thus, it can be applied when prolonged drug release is required. If short release times are needed, one may likely use other polymers with shorter degradation times.

Another limitation of our approach is the use of spin-coating to obtain the polymer layer. When the polymer solution (with species to be incorporated) is applied to the spinning substrate, the majority of the solution is lost and splashed around the substrate. Thus, to increase the efficiency of the process, other methods of film formation may be envisaged. We are currently intensively working on the application of our approach to other polymers, substrates, or deposition methods in order to efficiently control the preparation of polymer particles with desired properties.

## Supporting Information Available

Histogram demonstrating the hole diameter distribution within the spin-coated PLA layer; AFM of the annealed PLA film; histograms showing particle size distributions for the samples prepared from PLA chloroform solutions at varying concentrations (PDF)
