# Supplementary material for: Preparation of Surface-Supported Polylactide Spherical-Cap Particles
Source: Langmuir. 2022 Nov 17;38(48):14596–606. doi: 10.1021/acs.langmuir.2c01950 (PMC9730905; doi:10.1021/acs.langmuir.2c01950)
Supplement: Supplementary file 1 — la2c01950_si_001.pdf [file la2c01950_si_001.pdf]

Supporting Information

Preparation of surface-supported polylactide spherical-cap particles

Barbara Kuśmierz[1], Kamil Wysocki,[1,2], Maciej Chotkowski[1], Ilona Mojzych[1], Maciej Mazur [1,]*

[1]Department of Chemistry, University of Warsaw, Pasteura 1, 02-093 Warsaw, Poland

[2]Institute of Genetics and Animal Biotechnology, Polish Academy of Sciences, Postępu 36A, Jastrzębiec, 05-552 Magdalenka, Poland

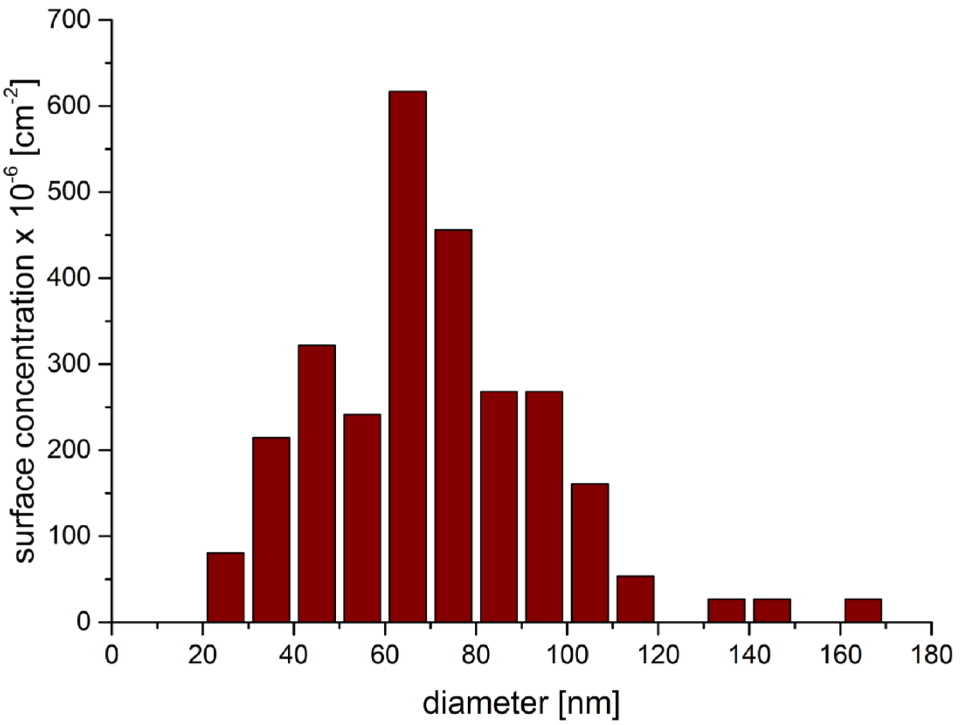

Figure S1. Histogram demonstrating the hole diameter distribution within spin coated polylactide layer (0.5% solution).

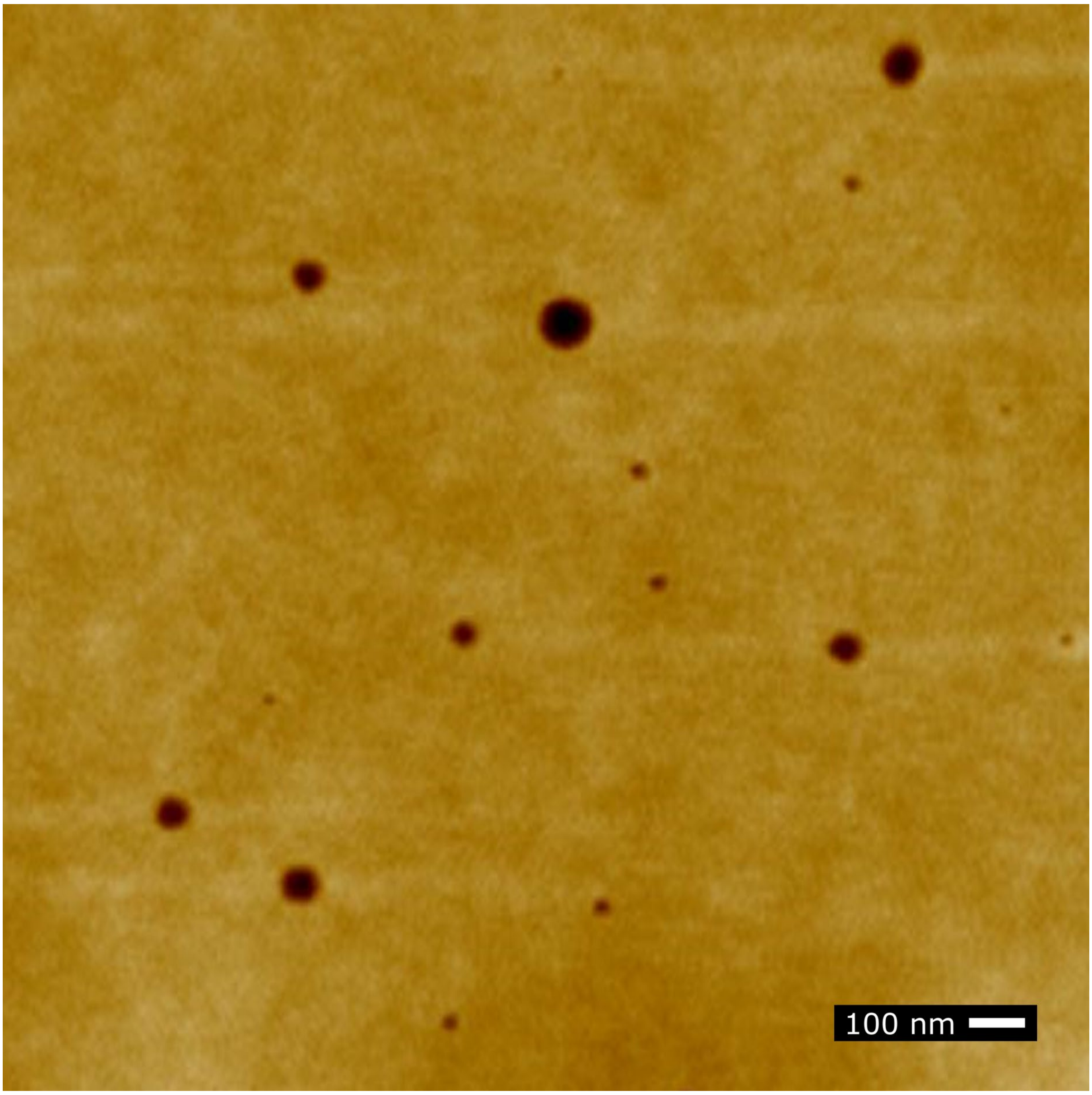

Fig. S2. Atomic force microscopy of polylactide film (spin coated from 0.5% chloroform solution) annealed at 180 ºC.

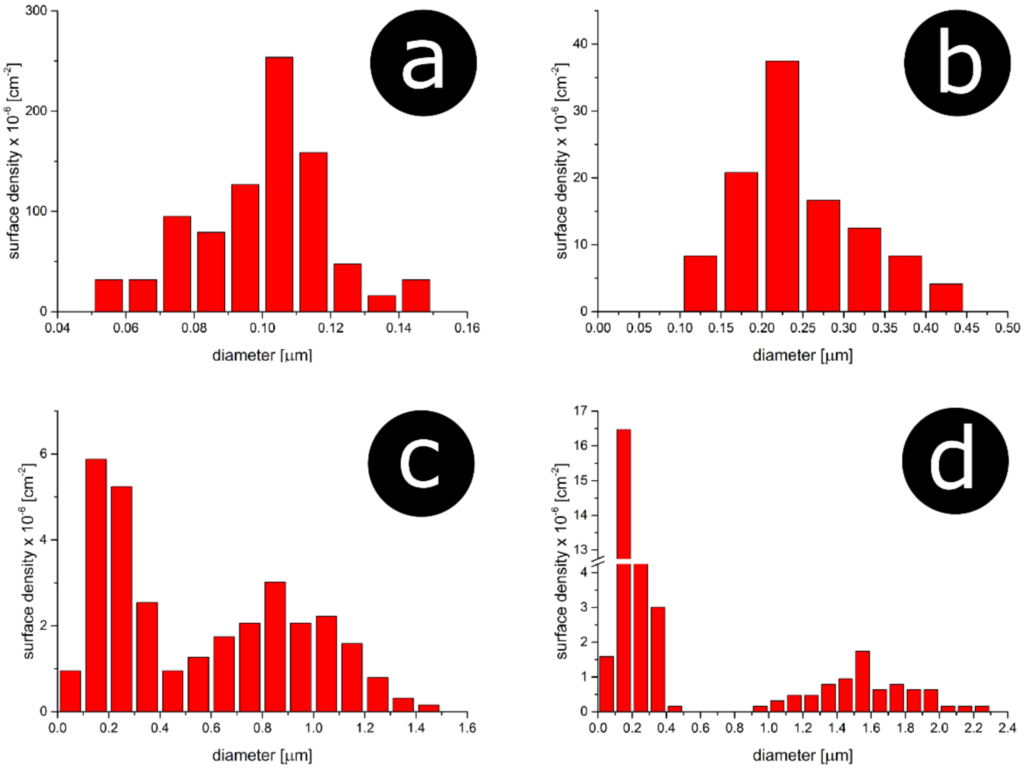

Fig. S3. Histograms showing particle size distributions for the samples prepared from PLA chloroform solutions: a) 0.1%, b) 0.2%, c) 0.5%, d)1%.
